# The *Caenorhabditis elegans* PTPN-22 protein tyrosine phosphatase non-receptor type 22 is required for neuronal migration

**DOI:** 10.17912/micropub.biology.002471

**Published:** 2026-10-31

**Authors:** Mark Yorio, Erik Lundquist

**Affiliations:** 1 Molecular Biosciences, University of Kansas, Lawrence, KS, United States

## Abstract

PTPN22 is a key negative regulator of T-cell activation in the vertebrate adaptive immune system. *Caenorhabditis elegans ptpn-22* encodes a molecule similar to vertebrate PTPN22, is expressed broadly, and regulates the formation of epidermal desmosomal adhesion complexes and cuticular molting. Work here shows that PTPN-22 is required for migration of the AQR and PQR neurons, a novel role for the PTPN22 family of molecules. Our results, along with those reported previously, suggest an ancestral role of the PTPN22 family in cell adhesion and migration, possibly reflected in its role in formation of the immune synapse in vertebrate T cells.

**Figure 1. ptpn-22 mutants displays AQR and PQR migration defects:** **
Figure 1. AQR and PQR migration defects in *ptpn-22* mutants.** A) A schematic of the QR and QL (QX) neuroblast lineage in the early L1 larva. The X axis is anterior to posterior, and the Y axis is time. A small “a” indicates the anterior daughter of a cell division, and a small “p” indicates the posterior daughter. Small grey circles indicate cells that undergo programmed cell death. B) UMAP coordinates for single-cell RNA-seq of the Q lineage indicating *ptpn-22* expression (see Methods). Cells are colored by expression level (purple), with gray indicating undetectable expression. C-D) The *lqIs244 [P gcy-32 ::cfp]* transgene was used to visualize the URX, AQR and PQR neurons. For each animal, Anterior (A), posterior (P), dorsal (D), and ventral (V) are indicated by arrows. The scale bar in C represents 10μm for both C and D. C) A *wild type* larva with *lqIs244 [ gcy-32 ::cfp]* expression. AQR is located in the anterior deirid ganglion near the posterior pharyngeal bulb (arrow). PQR is located posterior to the anus in the phasmid ganglion (arrow). The red box inset is a magnified view of PQR position posterior to the anus (dashed line and arrowhead). D) *ptpn-22 ( fd388 )* mutant L4 larvae with *lqIs244 [ gcy-32 ::cfp]* expression. In the top animal. PQR has failed to migrate posterior to the anus (arrow). The red box inset is a magnified view of PQR located immediately anterior to the anus (arrowhead and dashed line). The asterisk indicates the normal position of PQR. In the bottom animal, AQR has failed to migrate anteriorly to the anterior deirid and is instead displaced posteriorly behind the posterior pharyngeal bulb. The asterisk indicates the normal position of AQR. E) Quantification of AQR and PQR defects. The table indicates the positions of AQR and PQR in *wild-* type and *ptpn-22* mutants. The figure above the table shows the five positions along the anterior-posterior axis of the animal that were used to score AQR and PQR position. For each genotype, 100 animals were scored. The asterisks indicate that the AQR defects in the mutants were significantly less severe than those in *ptpn-22 ( fd388 )* and *ptpn-22 ( fd406 )* ( *p* < 0.03).

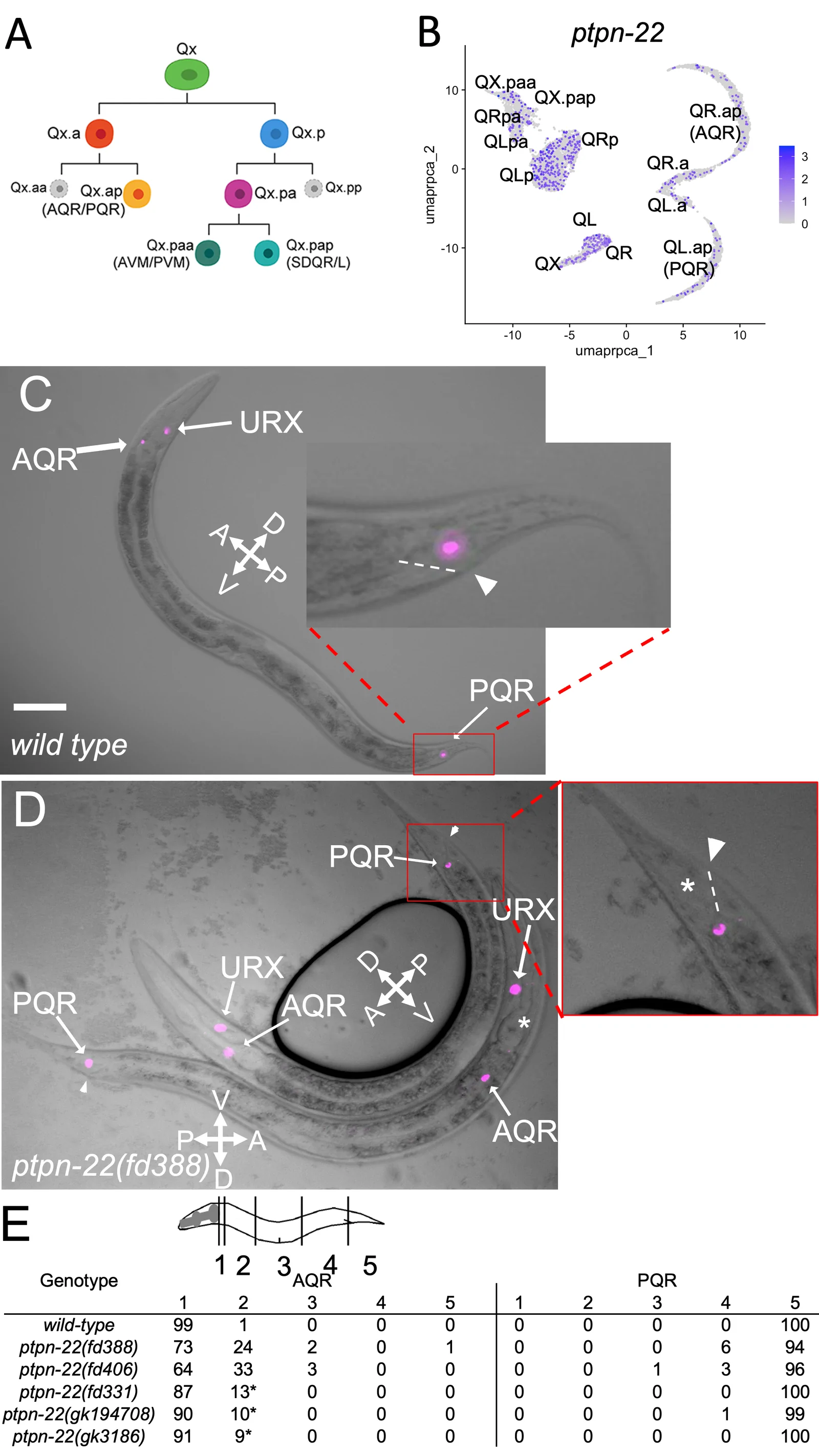



## Description

*C. elegans* PTPN-22 is an ortholog of the vertebrate protein tyrosine phosphatase non-receptor type 22 PTPN22 (Begovich et al., 2004; Binti et al., 2024; Cohen et al., 1999). PTPN22 has been investigated in mammalian systems for its roles in lymphoid and immune function (Bottini & Peterson, 2014; Cohen et al., 1999; Hill et al., 2002), notably suppression of T cell activation (Cloutier & Veillette, 1999) and viral immunity (Bevis et al., 2026; Orozco et al., 2024). In human populations, *Ptpn22* variants are associated with autoimmune disorders (Armitage et al., 2021; Gregersen et al., 2012). In humans, *Ptpn22* is expressed strongly in lymphoid tissues, with lower expression in other tissue types (Thul et al., 2017; Uhlen et al., 2015). In *C. elegans ,* mutations in *ptpn-22* have been shown to suppress cuticular molting defects due to mutations in NIMA-like kinases NEKL-2 and NEKL-3 , and to be associated with hypodermal adhesion complexes including hemidesmosomes (Binti et al., 2024).

Single-cell RNA sequencing of the Q neuroblast lineage shows that *ptpn-22* is expressed throughout the Q neuroblast lineages as they develop ( Figure 1A and B) (Teixeira et al., 2026). The bilateral Q neuroblasts, are derived embryonically and migrate in the L1 larva after hatching (Chapman et al., 2008; Middelkoop & Korswagen, 2014; Rella et al., 2016; Schild et al., 2023; Sulston & Horvitz, 1977). QR on the right migrates anteriorly over the V4 seam cell, and QL on the left migrates posteriorly over the V5 seam cell (Chapman et al., 2008). A similar pattern of cell division, programmed cell death, and neuronal differentiation results in three neurons on each side: AQR, AVM, and SDQR on the right, and PQR, PVM, and SDQL on the left. The QR descendant AQR migrate farthest anteriorly to the deirid ganglion near the pharynx ( Figure 1C ). The QL descendant PQR migrates the farthest posteriorly to the phasmid ganglion posterior to the anus ( Figure 1C ). Mutations that disrupt Q lineage migrations result in misplaced AQR and/or PQR neurons. Directional defects in migration ( *e.g.* anterior PQR migration or posterior AQR migration) result from mutations that affect the first Q cell migration and subsequent Wnt-mediated activation of the Hox transcription factor MAB-5 /Antennapedia (Josephson et al., 2016). Failure of migration ( *e.g.* AQR and PQR migrate in the correct direction but do not complete migration) can result from disruption of the migration substrate such as *epi-1 /Laminin-α5* mutation (Lang & Lundquist, 2021), or from mutation of downstream target genes of MAB-5 /Antennapedia (Jain et al., 2026; Jain & Lundquist, 2025).

We analyzed AQR and PQR migration in five *ptpn-22* alleles (Binti et al., 2024). All five alleles showed failure of complete AQR migration (9-33%) ( Figure 1D and E). PQR migration failure was noted in three of the five mutants (0-6%) ( Figure 1D and E). No directional migration defects were noted. Rather, AQR and PQR migrated in the correct direction but failed complete migration. Misplaced AQR were displaced posteriorly behind the pharynx, and misplaced PQR were displaced anteriorly, immediately anterior to the anus ( Figure 1D ). This phenotype is consistent with failure of the third migration of QL.ap/PQR posterior to the anus (Jain et al., 2026; Jain & Lundquist, 2025), and might also represent a late-stage failure of QR.ap/AQR migration (Middelkoop & Korswagen, 2014; Rella et al., 2016). *ptpn-22 ( fd388 )* introduces a premature stop codon in the second codon exon, and *ptpn-22 ( fd406 )* is a cysteine to serine missense mutation in the phosphatase catalytic domain (Binti et al., 2024). These two mutants displayed significantly stronger AQR migration defects compared to the other three alleles analyzed ( *fd331 ,* a frameshift; *gk3186* , a deletion of the first two coding exons; and *gk194708* , a premature stop codon in the second to last codon exon) (Binti et al., 2024; Thompson et al., 2013). Each of these is predicted to be a complete loss-of-function, and it is unclear why they have a significantly weaker phenotype than *ptpn-22 ( fd388 )* . This could be due to the genetic background of *ptpn-22 ( fd388 )* , or to retention of some function in *fd331 , gk3186 ,* and *gk194708* . The catalytically-dead *ptpn-22 ( fd406 )* could be significantly stronger because of a potential dominant-negative function ( *i.e.* the molecule might interact with binding partners leading to a non-functional complex). In sum, these results indicate that PTPN-22 is required for migration of the AQR and PQR neurons and defines a new function of the Ptpn22 family. While *ptpn-22* expression in the Q lineage is likely not controlled by MAB-5 /Antennapedia (Paolillo et al., 2024), the evidence here could suggest that PTPN-22 is required for the ability of AQR and PQR to migrate fully.

A balance of tyrosine phosphorylation and dephosphorylation is important in QR lineage migrations (Zhu et al., 2020): the SRC-1 /Src tyrosine kinase phosphorylates the guidance receptor MIG-13 to regulate actin polymerization in the direction of migration, and the receptor tyrosine phosphatase PTP-3 /LAR is responsible for mitigating this in the rear of the migrating cells. Src kinases are key substrates for PTPN22 activity in the immune system (Cloutier & Veillette, 1999; Wu et al., 2006). It is possible that PTPN-22 participates in this tyrosine phosphorylation balance underlying AQR and PQR migration via modulating cytoskeletal or cell adhesion functions. In any case, these results are consistent with an ancestral role of PTPN22 family members in regulating cell adhesion and migration.

## Methods

*C. elegans* strains were maintained and scored at 20°C. AQR and PQR were visualized using the *P gcy-32 ::cfp* transgene *lqIs244* (Chapman et al., 2008; Josephson et al., 2016) (Chapman et al. 2008; Josephson et al. 2016). AQR and PQR positions along the body were noted using a five-position scale as previously described (Jain et al., 2026; Jain & Lundquist, 2025; Josephson et al., 2016) (See Fig 1E). Statistical significance was determined using Fisher's Exact test.

## Reagents

The following *C. elegans* mutants and transgenes were used: LGII, *lqIs244 [P gcy-32 ::cfp];* LGIV, *ptpn-22 ( gk194708 ), ptpn-22 ( gk3186 ), ptpn-22 ( fd331 ), ptpn-22 ( fd388 ), ptpn-22 ( fd406 )* . The *tmC25* balancer ^27^ was used to isolate *ptpn-22* mutant chromosomes. The presence of *ptpn-22* mutations was confirmed by sequencing or PCR genotyping. The following strains were analyzed:

**Table:** 

Strain	Genotype	Origin
LE3885	*lqIs244 II *	(Josephson et al., 2016)
LE8131	*ptpn-22 ( gk194708 ) IV; lqIs244 II *	(Binti et al., 2024) and this work
LE8130	*ptpn-22 ( gk3186 ) IV; lqIs244 II *	(Binti et al., 2024) and this work
LE8128	*ptpn-22 ( fd331 ) IV; lqIs244 II *	(Binti et al., 2024) and this work
LE8127	*ptpn-22 ( fd388 ) IV; lqIs244 II *	(Binti et al., 2024) and this work
LE8129	*ptpn-22 ( fd406 ) IV; lqIs244 II *	(Binti et al., 2024) and this work
